# Estimating the optimal number of samples to determine the effective population size in livestock

**DOI:** 10.3389/fgene.2025.1588986

**Published:** 2025-06-03

**Authors:** Arianna Manunza, Paolo Cozzi, Paul Boettcher, Ino Curik, Christian Looft, Licia Colli, Johann Sölkner, Gábor Mészáros, Alessandra Stella

**Affiliations:** ^1^ Institute of Agricultural Biology and Biotechnology, National Research Council, Milan, Italy; ^2^ Animal Production Officer at Food and Agriculture Organization of the United Nations, Rome, Italy; ^3^ Department of Animal Science, University of Zagreb, Faculty of Agriculture, Zagreb, Croatia; ^4^ Institute of Animal Sciences, Hungarian University of Agriculture and Life Sciences (MATE), Kaposvár, Hungary; ^5^ Department of Animal Breeding and Husbandry, Hochschule Neubrandenburg—University of Applied Sciences, Neubrandenburg, Germany; ^6^ Dipartimento di Scienze Animali, Della Nutrizione e Degli Alimenti and BioDNA Centro di Ricerca sulla Biodiversità e sul DNA Antico, Università Cattolica del S. Cuore, Piacenza, Italy; ^7^ Institute of Livestock Sciences, BOKU University, Vienna, Austria

**Keywords:** effective population size, conservation, SNP arrays, simulation, small ruminants

## Abstract

Effective population size (*Ne*) is a key parameter in various biological disciplines, including evolutionary biology, conservation genetics, and livestock breeding programs. When applying genomic approaches to estimate *Ne* or other indicators of genetic variation, sample size is among the critical factors that directly affect the balance between cost and precision. In this study, we investigated the impact of sample size on *Ne* estimates by analyzing data from previous genotyping studies and simulations. Our results suggest that a sample size of 50 animals is a reasonable approximation of the “true” (“unbiased”) *Ne* value within the populations analyzed. While estimating the *Ne* value is an important starting point in population genetics, additional factors, such as the degree of inbreeding, population structure, and admixture, must be taken into account to obtain a comprehensive genetic evaluation and avoid misinterpretation. We conclude that linkage disequilibrium (LD)-based approaches are well suited for the estimation of *Ne* in livestock populations. However, careful interpretation of results is essential as current bioinformatics tools may introduce potential biases due to methodological assumptions, marker density, or population-specific factors.

## 1 Introduction

Effective population size (*Ne*) is widely considered to be an important parameter to be estimated in several contexts of biological concerns such as evolutionary and conservation biology and breeding programs (Waples, 2024; 2025). *Ne* quantifies the magnitude of genetic drift and inbreeding of populations. Originally introduced in the 1930s (Wright, 1931), the initial theory was based on idealized panmictic population at drift–migration equilibrium, thus considering genetic drift as the only factor acting on the allelic frequencies. The concept was progressively extended to account for the other evolutionary forces influencing *Ne* in real populations. Methods were developed to predict *Ne* at different spatial and timescales and under various demographic scenarios (Wang et al., 2016). *Ne* can be estimated using demographic, pedigree, and genomic data sources. When using demographic information, *Ne* is generally calculated based on the anticipated change in inbreeding per generation (∆F), considering the number of breeding males and females as well as the variance in family size. With pedigree data, *Ne* is determined from the inbreeding coefficients over generations, again using ∆F as the basis. The growing availability of advanced genomic technologies enabled the estimation of *Ne* from genetic markers, which is particularly useful if no pedigree information is available. In genomic data, the three primary methods for estimating *Ne* utilize (i) the temporal method based on the change in inbreeding coefficient ∆F, which reflects the rate of genetic drift; (ii) the rate of coancestry, which measures the increase in genetic relatedness among individuals over time; and (iii) the degree of linkage disequilibrium (LD) between neutral loci, which provides insights into historical and contemporary population structure and size. Many efforts have been made to develop statistical methods and approaches that allow the computation of *Ne* from genomic data (Beichman et al., 2018; Novo et al., 2022; Novo et al., 2023; Ryman et al., 2019; Santiago et al., 2024; Wang et al., 2016; Waples and Do, 2010). These methods have been applied to estimate both contemporary (recent) and historical *Ne* with different inference methods, methodological approaches, and applications (Hare et al., 2011; Nadachowska-Brzyska et al., 2022). A commonly used definition for contemporary *Ne* is the effective size for the period of time covering the sampling, for which the calculation is based on the linkage disequilibrium (LD) observed using unlinked markers. These estimates find a practical application in conservation because they can offer useful management advice (Waples, 2024; Waples, 2025). Historical *Ne*, calculated using linked markers, is related to past demographic events and is relevant in phylogeographic reconstruction of both wild and domesticated populations (Novo et al., 2023). Many of the bioinformatics tools that implement the LD method (Ne_LD_) are specific for one of the two inferences, either contemporary or historical, but often with a slight difference in the time in terms of generations for which they provide information (Nadachowska-Brzyska et al., 2022). In addition, the term “recent” can refer to different time points within the interval of the evolutionary time we are considering. For livestock species, the possibility of estimating changes in population size in the recent past is relevant in conservation of genetic diversity and particularly in selecting samples for banking of germplasm material. The application of genomic tools in livestock is becoming a conventional practice, especially in commercial breeds, due to the low costs of genotyping. However, for local breeds or breeds that are the target of conservation strategies, the trade-off between cost of genomic analysis and the potential economic returns makes its application less relevant from an economic point of view (Bruford et al., 2015). Conservation programs are often underfunded (White et al., 2022) and, therefore, preclude genotyping a large number of animals. To render the utilization of genomics tools effective in practice, it is necessary to find a compromise between the number of sampled individuals and the precision of the estimate of *Ne* and other parameters that need to be evaluated. The aim of this study was to assess the optimal number of individuals to be genotyped to obtain the best approximation of *Ne*. Data from two livestock species (sheep and goats) were used. We chose three sample sizes and compared the results from simulated and empirical data. We used SNP genotypes from public databases of both local and transboundary goat and sheep breeds, applying the Ne_LD_ method implemented in NeEstimator v.2 (Do et al., 2014). The *Ne* estimates based on demographic and pedigree information were available for some of the breeds included in the dataset, and we compared them with our genomic estimates. In addition, we simulated a one sheep population and calculated its effective size under six different scenarios to explore the effect of some demographic changes and other evolutionary forces (e.g., the selection scheme).

## 2 Materials and methods

### 2.1 Characteristics of the analyzed breeds and their genotyping data

Specifically, we used publicly available genotype data retrieved for two goat breeds (Murciano-Granadina and Alpine) and two sheep breeds (Churra and Tibetan). For the goat and sheep populations, the markers’ positions were assigned based on the caprine genome assembly ARS_v1.0 and the ovine genome assembly Oar_v3.1, respectively, using the SNPchiMp v.3 database (Nicolazzi et al., 2015) and by using a series of custom scripts developed in the context of the SMARTER project (https://smarterproject.eu/) (Cozzi et al., 2024). For more information about samples retrieved in the SMARTER database, see the following link: https://webserver.ibba.cnr.it/smarter/about.

The Spanish Murciano-Granadina (MG) goat breed was created in 1975 from two breeds: Murciana and Granadina. According to the most recent census, the MG breed numbers more than 100,000 individuals (Guan et al., 2021). The MG is typically raised in semi-intensive conditions, primarily for cheese production (Delgado et al., 2018), and one of its main features is its extraordinary adaptation to harsh climatic conditions (Spanish Ministry of Agriculture, Fisheries, and Food). For the MG population, the data comprised 1,040 female goats from 15 farms located in the autonomous region of Andalusia (Spain) and genotyped with the Goat SNP50K Illumina BeadChip (Luigi-Sierra et al., 2022).

The Alpine goat is a medium- to large-sized breed known for its very good milking ability. The breed originated in the French Alps and is now one of the most popular dairy breeds around the world. More than 450,000 individuals are recorded in the local census in France alone. Genotype data for 279 individuals genotyped with the Goat SNP50K Illumina BeadChip were retrieved in the SMARTER database (Cozzi et al., 2024), and they were originally genotyped in the framework of the AdaptMap project (Stella et al., 2018), whose samples were from France, Switzerland, and Italy.

The Spanish Churra is an autochthonous dual-purpose breed. Milk production of Spanish dairy sheep breeds has been the subject of intensive breeding programs, and the Churra has experienced a 15%–20% increase in yield during the last 25 years (Churra Breeding Association web, http://www.anche.org). The current population size in Spain is over 150,000 animals. Genotypes (Illumina OvineSNP50 BeadChip) for 270 animals were retrieved in the SMARTER database (Cozzi et al., 2024) and from the study by Kijas et al. (2012).

The Tibetan sheep is among the most common breeds in northwestern China, with more than 23 million animals distributed throughout the Qinghai–Tibet plateau. Originating from northern Chinese ancient sheep ∼3,100 years ago, Tibetan sheep gradually evolved into different ecotypes depending on geographic conditions. Their adaptation to harsh environments makes them an important resource for the economic and social development of the local people. Our study included 820 individuals characterized by Illumina OvineSNP50 BeadChip and whole-genome sequencing retrieved in the SMARTER database (Cozzi et al., 2024) and originally from the study by Wang et al. (2016).

### 2.2 Procedure for empirical and simulated data

Genotype data were edited following FAO recommendations (Ajmone-Marsan et al., 2023) using PLINK v1.9 and v2 (Chang et al., 2015). Supplementary Figure S1 illustrates the workflow for the quality control (QC). To be consistent, we applied the same setting for the pruning procedure (QC) keeping the correlation coefficient between SNP pairs (r2) threshold to 0.5, thus removing markers in high linkage disequilibrium (LD), as the loci are assumed to be unlinked. The QC procedure left 214, 895, 233, and 659 animals and 35,375, 45,487, 18,708, and 35,529 markers for Alpine, MG, Churra, and Tibetan breeds, respectively. This range of SNP numbers aligns with the marker densities commonly reported in recent genetic diversity research. For each replicate, *Ne* estimates were obtained using two LD-based methods, as implemented by NeEstimator v2.1 (Do et al., 2014). In addition, as a basis for comparison, *Ne* was calculated for each breed by using the entire dataset (post quality control) of available animals and marker information. All the analyses were performed by applying the Nextflow (Di Tommaso et al., 2017) pipeline (v0.2.1), which is purposely developed and publicly available at cnr-ibba/nf-neestimator. The workflow automated the following: (i) the random sampling of individuals, (ii) the conversion from binary files to the GENEPOP format (PLINK v1.9 and PGDSpider v2.1.1.5 (Chang et al., 2015; Lischer and Excoffier, 2012), and (iii) the *Ne* estimation procedure and the LDNe procedure in NeEstimator. The Pcrit parameter was set in the program to screen out alleles at the frequency <0.02 because this criterion provides a generally good balance between maximizing precision and minimizing bias (Waples and Do, 2010). We also applied the sample size correction before analyzing data, thus ensuring that the estimates are more accurate even when the sample size is small, as described by Waples (2006).

The harmonic mean as implemented in the program was used. This approach is standard because the harmonic mean places greater weight on smaller population sizes. The harmonic mean provides a more accurate representation of long-term genetic variation than the arithmetic mean, particularly in populations that experience bottlenecks or fluctuations in size. The program also provides a fixed-inverse variance-weighted harmonic mean correction for missing data for the linkage disequilibrium and temporal methods. Simulation was used to complement the results obtained with the real population data. Simulations were performed using the QMSim 2.0 program (Sargolzaei and Schenkel, 2009). Six scenarios mimicking small ruminant populations were simulated: POP1 = selection based on phenotype, POP2 and POP2_cd_h = selection design (sd) with selection based on estimated breeding values (high selection intensity for both) plus culling design (cd) high for POP2_cd_h, POP3 = same as in POP1 but with the application of a recent population bottleneck, POP4 = POP1 but with a recent expansion event, and POP5 = constant population size and random mating (contribution). Historical population is simulated based on the forward-time approach, and the program can only simulate a single historical population. A full description of the setting is available in the Supplementary Material. In brief, for the bottleneck event in our simulation, we modeled a classic bottleneck scenario characterized by a sudden reduction in the population size from 1,000 to 200 individuals at generation 70, followed by a prolonged bottleneck phase lasting 30 generations, before a moderate recovery. This setup allowed us to explore the lasting effects of a rapid-onset, long-duration demographic contraction on *Ne* estimation. We simulated a population expansion in a recent population. In the expansion scenario, the historical population size remained stable at 420 individuals for 200 generations, and the forward simulation began with a modest number of founders (420 individuals). Over 10 generations, a gradual population increase was allowed through controlled reproduction (litter size = 2), modeling a slow and recent expansion. This scenario enabled us to assess how limited growth over a short time frame affects *Ne* estimates under LD-based methods.

The six scenarios shared parameter settings for heritability (0.20), phenotypic variance (1.0), litter size (1), and the proportion of male progeny (0.5). We simulated genetic data for populations of fixed size (N = 2,400 individuals), with 26 chromosomes. As with the real data, we randomly selected 20, 50, and 100 individuals for each of the 100 iterations. *Ne* was estimated using the same methods as for the real dataset. Estimates were based on the whole set of 52,000 simulated SNPs, rather than approximately 35,000 SNPs (post-filtering) as in the real-population data. Supplementary Figure S2 summarizes the workflow followed in this study. For each population, three sampling sizes were investigated: 20, 50, and 100 animals, sampling individuals without replacement. One hundred replicates were applied for each scenario (i.e., breed * sampling size).

## 3 Results

### 3.1 Estimating *Ne* from empirical data

**FIGURE 1:**
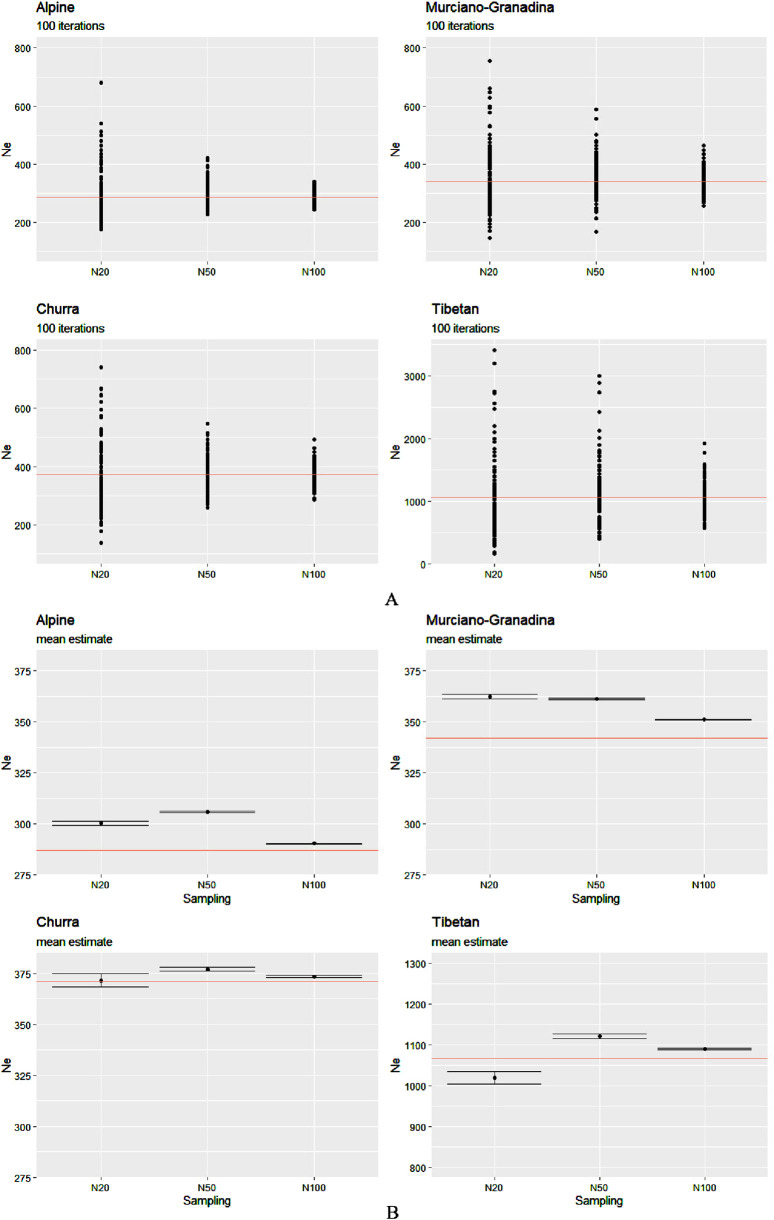
*Ne* estimates calculated for the four breeds of the two species over a range of sizes: 20, 50, and 100 animals. **(A)** Solid circles represent the estimates from 100 independent iterations and **(B)** each black point corresponds to the mean value of 100 estimates with the CI. The “true” effective size, the value of which was calculated based on the entire dataset, is indicated by the red horizontal line.

For each sample size, the estimates of the *Ne* derived from LD after 100 iterations and their descriptive statistics (e.g., mean, standard deviation, and confidence intervals (CIs)) were obtained and are illustrated in Figures 1A, B; Supplementary Table S1.

As anticipated, the estimate for N100 consistently demonstrated the highest accuracy, yielding values closest to the “true value” across all species and breeds. In contrast, the estimates derived from N20 often deviated more substantially, typically showing a tendency toward overestimation. This trend was accompanied by broader CIs for N20, indicating increased uncertainty at lower sampling sizes. Notably, several outliers were present in both the N20 and N50 estimates for Tibetan sheep; however, this pattern was markedly more pronounced at N20 across all breeds.

Although sampling at N50 resulted in slight overestimations, it provided a reasonably close approximation to the full dataset, which was comparable in precision to that of N100. A closer inspection of mean *Ne* values revealed consistent overestimation in the two caprine breeds, with a particularly pronounced peak at N50 in Alpine. In the sheep breeds, overestimation at N50 and N100 was minimal. An exception to the general overestimation trend was observed in the Tibetan population at N20, where the mean estimate of *Ne* fell below the reference value. This underestimation may reflect the sensitivity of *Ne* estimators to small sample sizes in structured populations, particularly when rare alleles or subpopulation structures are underrepresented due to limited sampling. Such bias is consistent with the demographic complexity and potential substructure of the Tibetan breed. Interestingly, although the mean *Ne* estimate for N20 in Churra hovered around the true value (∼370), the much wider CI suggests a lack of precision and robustness at that sample size. In contrast, both N50 and N100 estimates consistently exhibited narrow CIs across all populations, reflecting a higher degree of precision and reliability in the *Ne* estimation at increased sampling depths.

### 3.2 Estimates of *Ne* for simulated data

**FIGURE 2:**
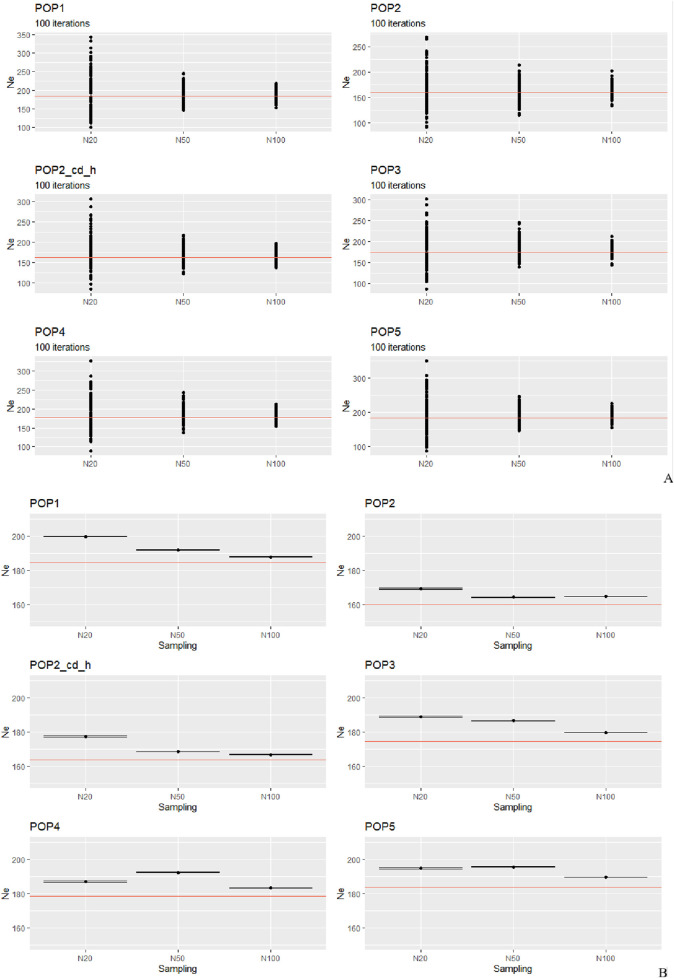
Simulated scenarios. The *Ne* estimates of 20, 50, and 100 subsampled individuals for every population are plotted against the effective sizes calculated for the whole population (indicated by the red horizontal line whose value can be retrieved in Supplementary Table S2). **(A)** Solid circles represent the estimates from 100 independent iterations and **(B)** each black point corresponds to the averaged value of 100 estimates. The black point shows the harmonic mean of 100 estimates.

The estimation of *Ne* for the simulated populations returned interesting evidence (Figures 2A,B).

By plotting the 100 iterations (Figure 2A), we observe a pattern similar to that of the real dataset: N20 produces the most biased and variable estimates, whereas N50 and N100 exhibit fewer outliers and estimates that fall more closely around the true value. Examining the average estimates (Figure 2B), POP1 and POP3 share the same selection design based on phenotype but differ in their demographic history. Notably, POP3 has experienced a severe population reduction (from 1,000 to 420 sheep) due to a bottleneck event. Although the N100 estimate in all cases is the closest to the “true one,” the difference between N50 and N100 is of only a few animals. POP2 and POP2_cd_h share a constant size and the **sd** based on estimated breeding value but, in the second case, the cd based on phenotype is high (see materials and methods for details). As we can see, although in both cases the estimate for N20 gives the worst result, we obtained a different result for N50 that performs better than N20, but in POP2, it is also somewhat closer to the true value than N100. POP4 and POP5 have two opposite situations, characterized by different sd and demographic histories. In POP4, which has experienced an increase in its population size, the method provides better performance for N100 followed by N20, whereas N50 slightly overestimates the real value. However, POP5, which corresponds to an ideal scenario of constant population size and random mating, shows just a little difference between N20 and N50, with a CI for N20 estimate being somewhat bigger. Although N100 plots demonstrate that with a subsampling of 100 animals the program performs much better, on comparing N50 to N20, we noticed that when using N50, the linkage disequilibrium-based *Ne* estimator performed reasonably well, giving more uniform results. Overall, the harmonic mean estimate from 100 simulations was usually close to the true *Ne* when the sample size was N100 in our simulated and natural datasets, and the estimates were often severely biased upward when the sample size was equal to 20.

## 4 Discussion

Genomic methods are routinely used to estimate contemporary *Ne* with preferences toward LD-based methods, especially when pedigree data are not available. In our work, we apply LD-based methods to analyze *Ne* in two livestock species coming from different farming and breeding conditions as well as different natural environments. The estimated *Ne* in our real-life populations was approximately ∼350 animals in MG and Churra, whereas it is lower for Alpine (∼285) and much higher for Tibetan sheep (>1,000). Only a few previous studies that aimed to infer the “historic *Ne*” of the same breeds included in this research were available. Thus, comparison with those studies was difficult as the *Ne* calculation was based on pedigree data (Oliveira et al., 2016) or using the LD method for the historic *Ne* (Chitneedi et al., 2017; Colli et al., 2018; García-Gámez et al., 2012; Liu et al., 2021). Indeed, in this last case, using the LD method, the most recent estimate referred to the last 13 to 5 generations, which corresponds to approximately 60–20 years before the sampling, making the comparison with our outcomes more difficult. Moreover, the method used to assess the historic *Ne* trends is based on different assumptions (Waples, 2024; Waples, 2025), resulting in different estimates. However, in our study, we observed generally higher *Ne* estimates, especially for Churra and Tibetan sheep, than those obtained in previous studies (García-Gámez et al., 2012; Chitneedi et al., 2017; Liu et al., 2021), whose estimates were approximately 128 and 160 (Churra) and 250 animals (Tibetan). The aforementioned studies of Churra breed differ for the total number of markers and their density along the genome: Garcia-Gamez et al. (2012) employed a medium-density 50K chip, whereas Chitneedi et al. (2017) used high-density imputed data. This may be the reason for obtaining different *Ne* estimates even if the datasets partially overlapped. Those estimates were also lower than our findings for the same breed, as stated before, and this can be associated with two more sources of bias: i) sample design (the animals included in those studies were highly related) and ii) the quality control procedure prior to carrying out the analysis (the dataset was not filtered for LD). Both factors contribute to produce estimates of *Ne* that are downwardly biased because of excess LD caused by linkage rather than drift (Sved et al., 2013). Our QC procedure produced datasets without these two sources of bias: in particular, from the kinship relatedness analysis after QC (Supplementary Figure S3), we can observe a distribution of kinship coefficient that suggests small internal relatedness. There are also previous investigations based on other sheep and goat breeds that reported downwardly biased *Ne* estimates, and all of them share the inclusion of linked loci in their datasets (Vlaic et al., 2024; Becker et al., 2024; Prieur et al., 2017; Liu et al., 2017), thus providing less accurate estimates. Considering the high census size of MG (Delgado et al., 2018) of over 100,000 individuals raised all over Spain and the currently ongoing official breeding and conservation programs, our estimate for this breed agreed reasonably well with those from other local Spanish goat breeds such as Bermeya and Malaguena (∼200 individuals (Colli et al., 2018)) and are consistent with its reportedly good levels of genetic diversity (Oliveira et al., 2016). MG, with minimal relatedness (Supplementary Figures S3, S4), yielded consistent and accurate *Ne* estimates even at small sample sizes. The most recent estimate found in literature for Alpine was approximately 150 animals (Santiago et al., 2020), which is quite low. This difference with our estimate is most likely due to the different methods applied as the historical *Ne* is based on linked markers for the demographic reconstruction (Novo et al., 2022). This transboundary breed has a high census size, is under intensive selection, and is widely employed in breeding programs to improve the milk production performance of less productive (local) breeds. However, a large census size does not necessarily correspond to a high *Ne*, particularly under intensive artificial selection, which is known to reduce *Ne* due to factors such as strong selection intensity, reduced sire diversity, and unequal parental contributions (Waples, 2016). This is exemplified by Holstein cattle, where intensive selection has led to low N*e* despite a very large population (Makanjuola et al., 2020). In contrast, the moderately higher *Ne* we observed for Alpine may reflect differences in the breeding structure and strategies applied in this breed potentially, including less centralized selection, more diverse use of breeding animals across regions, or continued gene flow among subpopulations. These factors could contribute to retaining more genetic diversity than in more intensively selected or closed populations, thus supporting a more favorable *Ne* outcome than might otherwise be expected. In addition, the combination of these factors and the possible effect of the population structure (Supplementary Figures S3, S4) due to the inclusion of genotypes from three different countries in our experimental design may have contributed to this difference. Conversely, there are several factors that can affect both past and contemporary *Ne* inference, such as selection and migration as well as strong changes in the population size (bottlenecks and population expansion) and population structure (Waples, 2024; Waples, 2025). Novo et al. (2022) addressed the question of whether natural selection can bias estimates of *Ne* that assume selective neutrality, and they found that the historical *Ne* is almost unaffected by selection; this finding reasonably allows us to conclude that contemporary *Ne* also should show negligible or no bias due to selection (Waples, 2024; Waples, 2025). Except for Tibetan sheep, the breeds we investigated are subject to specific artificial selection breeding programs. Notably, the intensive selection of Spanish sheep breeds such as Churra for milk production is relatively recent, having begun only 3–4 decades ago (Manunza et al., 2016). During this period, genetic exchanges between dairy and non-dairy populations may have also occurred, potentially obscuring the detectable effects of confounding factors. Furthermore, these breeds have undergone demographic changes, including a decline in population size, as indicated by previously cited studies. The estimates of *Ne* in MG and Churra reflect the retainment of an effective degree of genetic variability because of the establishment of recent balanced selection-conservation programs (Delgado et al., 2018). Microsatellites represent a valuable source of information for assessing both genetic diversity and *Ne.* Previous studies employing similar markers, specifically microsatellites (SSRs or STRs), in three Spanish local ruminant populations—the Pajuna cattle, Payoya goat, and Merino de Grazalema sheep (Cervantes et al., 2011)—as well as in other local Spanish (Álvarez et al., 2008) and Indian sheep breeds (Punuru et al., 2025), reported lower estimates than those obtained in the present study. These discrepancies may be attributed to the conservation status of the populations examined in those studies, all of which involved rare breeds. As noted by the respective authors, *Ne* values were likely underestimated in their analyses, whereas our estimates appear to be slightly inflated. Therefore, when feasible, the integration of multiple types of genetic markers may be recommended to improve the accuracy of *Ne* estimations, particularly in populations of conservation concern. For the Tibetan sheep, our estimates were very large, and this is probably due to the huge census size and the presence of the population substructure, as we can observe in Supplementary Figure S3. N20 underestimates *Ne*, likely due to the presence of closely related individuals in the small sample. The population was sampled over a wide area of China, that is, the Qinghai plateau region (Li et al., 2021), where many local populations and ecotypes are present. In subdivided populations, the estimate of *Ne* can reflect the average changes in allele frequencies and inbreeding in the metapopulation except when one (or more) subpopulation has more influence with respect to another one. In this case, the estimate could be likely more related to a process specific to local subpopulations dynamics rather than to the metapopulation “as a whole,” resulting in a “larger” or “smaller” *Ne* than expected (Ryman et al., 2019). In addition, when the ratio of Ne/N is very large, the uncertainty associated with the estimate will usually be very large (Wang et al., 2016; Waples, 2016) because large *Ne* produces a very weak drift signal. Waples, (2016) demonstrated that with large *Ne* and only a moderate sampling of individuals (such as N20 and N50 in our study), many estimates were much too low, many were much too high, and very few were close to the true value. Another general consideration is that in agreement with our overall results, simulations showed that LD-based estimators are strongly biased when the sample size is small (England et al., 2006). Waples (2006) already demonstrated that demographic changes can play an important role while assessing *Ne* from empirical data: following a bottleneck, the signal generated by the increased new LD arising from the recent reduction in *Ne* blurs the higher background values of *Ne*. Indeed, following a population expansion, the drift signal is still too small for the new *Ne* to be closely approximated to the expected estimate, requiring more generations after the event for a stronger drift signal to be detected with the methods currently available (Waples, 2005).

One of the scenarios that we tested (POP4, Figures 2A,B) included a gradual expansion and spanned only a few generations. In such cases, the estimation of *Ne* is especially sensitive to the sample size. Our results indicate that although N20 occasionally produced estimates closer to the true value, this occurred inconsistently and appears to result from random sampling effects, particularly under recent expansion, where residual LD can be more variable. However, N20 was also associated with a higher variance of estimates, reflecting its greater susceptibility to stochastic sampling noise and the inclusion of closely related individuals. This inconsistency reduces its reliability, particularly for empirical studies where replicate testing is not feasible. In contrast, N100 consistently yielded the least biased and most stable estimates, but such a sample size may be impractical in many real-world livestock studies due to budgetary or logistical constraints. Notably, N50 emerged as the most balanced compromise, offering substantially reduced dispersion compared to N20, while still being feasible for routine application in conservation and breeding programs. In more detail, slight upward bias in N50 can be related to the slow and recent nature of the demographic increase of population. With a moderate sample size (N50), the low level of LD in the expanded population is harder to capture accurately in comparison to N100, leading to a slight overestimation of *Ne*. In contrast, the smaller sample size (N20), although more affected by sampling variance, sometimes captured higher levels of residual LD, yielding slightly more accurate *Ne* estimates. This suggests that in recent expansion scenarios, random sampling effects can by pure chance improve *Ne* estimation accuracy in small samples by mitigating LD decay bias. The sampling issue also regards the assumption of randomness, where each individual has the same chance of being sampled. In nature, perfectly random sampling is usually difficult to achieve, and the most common sampling bias occurs when close relatives are sampled at higher rates. One possible solution can be to exclude very related individuals (e.g., siblings). The underlying problem is that pruning for close relatives can also lead to biased *Ne* estimates as the incidence of relatives is a fundamental part of the genetic-drift signal, and without additional information from the pedigrees, it is impossible to know how many individuals to remove to approximate a random sample (Waples, 2024; Waples, 2025). This notable difference in the performance of the *Ne* estimation depending on the sample size is of particular importance in both breeding and conservation programs, where maintaining high levels of genetic diversity and keeping inbreeding low are important (Ryman et al., 2019). For practical applications, the most important considerations regarding the estimation of contemporary *Ne* are the following: based on our results, despite the presence of potential confounding factors, the representative sample size should be N100. Through the graphic comparison between the patterns presented in Figures 1, 2 for these two datasets (natural and simulated populations), we have come to the same conclusion. We observed that all means are overestimations (except for Tibetan), especially for smaller sample sizes. To reduce this bias, we applied the bias correction described by Waples (2006). This correction accounts for the fact that small sample sizes can inflate *Ne* estimates by leading to artificially low LD values when too few individuals are sampled. A second option is to use a larger sample size, but this is often less viable in real-world applications, especially when dealing with livestock species and breeds that are the target of non-profit-making projects. Indeed, finding a cost-effectiveness balance is a priority for most of the conservation and breeding programs. Addressing 100 animals would be unfeasible for the available resources of most laboratories, projects, and biobanks. This rationale can also support our conclusion for N50 to be the best compromise to reach this balance (the *Ne* values obtained using N50 overall showed that this sample size is a reasonable approximation to the true value). When using the *Ne* of a local population in designing its diversity management program, it is necessary to complement the results with other information and analyses such as the level of inbreeding, population structure, admixture, the inbreeding depression in fitness related traits, the genetic load, and a more comprehensive demographic study. Many populations lack these important clues and, under such circumstances, the outcomes obtained from this estimator could be more difficult to interpret. Finally, even if the next-generation sequencing approaches provide interesting opportunities, this method of recent *Ne* inference does not improve the chance of a more reliable estimation by simply increasing the number of markers: indeed, little extra precision is gained by using more than a few thousand variants.

## 5 Conclusion

In conclusion, our study highlights the usefulness and limitations of LD-based methods for estimating contemporary *Ne* in livestock populations, particularly in the absence of pedigree data. Our estimates, which were generally higher than those from previous studies, reflect the influence of factors such as marker density, sample size, population structure, and recent demographic history. Breeds such as the Churra and MG show *Ne* values consistent with active breeding and conservation programs, whereas the very high *Ne* observed in Tibetan sheep likely reflects both its vast census size and population substructure. Populations with higher internal relatedness or substructure (e.g., Tibetan but also Alpine) displayed greater sampling sensitivity in *Ne* estimates. Our findings reinforce the importance of using adequately sized and well-designed samples to minimize bias in a context of conservation programs for local breeds (Hampton et al., 2019; Bruford et al., 2015; White et al., 2022). Therefore, rather than focusing on minimal differences in average N*e* values between sample sizes, which can fluctuate due to stochastic effects or specific demographic scenarios, we recommend N50 for its favorable balance among estimation precision, logistical feasibility, and robustness to sampling variance. This makes it especially suitable for application in livestock management programs where genomic monitoring is integrated into decision-making but resources for sampling may be limited. Nonetheless, these estimates should be interpreted cautiously, complemented by other genetic indicators, and supported by the comparison of *Ne* estimates calculated using high- or medium-density SNP data and microsatellites marker. LD-based *Ne* estimation, although not novel, remains a valuable tool when used with appropriate design and context. Although further scenarios and methods can still be explored to improve the accuracy and applicability of N*e* estimation, new perspectives are suggested in this study for future and more complex investigations.

## Data Availability

Publicly available datasets were analyzed in this study. These data can be found here. The dataset supporting this study can be found in the SMARTER project (https://smarterdatabase.readthedocs.io/en/latest/index.html) (Cozzi et al., 2024).
