# Supplementary material for: Estimating the optimal number of samples to determine the effective population size in livestock
Source: Front Genet. 2025 Jun 3;16:1588986. doi: 10.3389/fgene.2025.1588986 (PMC12170570; doi:10.3389/fgene.2025.1588986)
Supplement: Supplementary file 1 [file Table1.docx]

| Alpine | | | | | |
| --- | --- | --- | --- | --- | --- |
| Sample size | Mean | SD | parametric_ci_low | parametric_ci_high | |
| TOT (*Ne*=287) |  |  |  |  | |
| Ne20 | 300.37 | 88.49 | 299.29 | 301.45 |  |
| Ne50 | 306.10 | 39.45 | 305.72 | 306.47 |  |
| Ne100 | 290.46 | 20.72 | 290.28 | 290.64 |  |
| Murciano-Granadina | | | | | |
| Sample size | Mean | SD | parametric_ci_low | parametric_ci_high | |
| TOT (*Ne*=342) |  |  |  |  | |
| Ne20 | 360.37 | 141.03 | 359.12 | 361.65 |  |
| Ne50 | 356.53 | 68.85 | 356.13 | 356.93 |  |
| Ne100 | 350.50 | 40.38 | 350.30 | 350.69 |  |
| Churra | | | | | |
| Sample size | Mean | SD | parametric_ci_low | parametric_ci_high | |
| TOT (*Ne*=371) |  |  |  |  | |
| Ne20 | 371.67 | 123.16 | 368.51 | 374.88 |  |
| Ne50 | 377.08 | 60.80 | 376.00 | 378.16 |  |
| Ne100 | 373.52 | 37.50 | 372.98 | 374.07 |  |
| Tibetan | | | | | |
| Sample size | Mean | SD | parametric_ci_low | parametric_ci_high | |
| TOT (*Ne*=1068) |  |  |  |  | |
| Ne20 | 1019.03 | 644.85 | 1003.61 | 1035.10 |  |
| Ne50 | 1120.97 | 499.99 | 1115.45 | 1126.56 |  |
| Ne100 | 1090.58 | 286.71 | 1088.23 | 1092.93 |  |

Supplementary Table 1. Summary statistics for the four breeds.
