# Supplementary material for: Estimating the optimal number of samples to determine the effective population size in livestock
Source: Front Genet. 2025 Jun 3;16:1588986. doi: 10.3389/fgene.2025.1588986 (PMC12170570; doi:10.3389/fgene.2025.1588986)
Supplement: Supplementary file 2 [file DataSheet1.docx]

**Supplementary Information**

Simulations were performed using QMSim 2.0 program (Sargolzaei & Schenkel, 2009), contrasting six possible scenarios. We set the parameters as follow: i) first scenario (POP1) with a selection design (sd): phen/low and a constant population size; ii) second scenario (POP2) with a sd: ebv/high and a constant population size; iii) third scenario (POP2_cd_h), same as POP2 but with culling design (cd) set to high (h); forth scenario (POP3) with a sd: phen/low and a bottleneck effect; iv) fifth scenario (POP4) with a sd: phen/low and a population expansion; v) sixth scenario (POP5) with a sd: rnd and a constant population size. The six scenarios shared some parameters setting: the Heritability value (h2) set to 0.15, QTL heritability (qtlh2) = 0.005, Phenotypic variance (phvar) = 1.0, litter size (ls) = 2 and the proportion of male progeny (pmp) = 0.5. From the total population generated by the program, that included 2400 sheep, 52,000 SNPs and choosing a LD mating model “monogamy”, we randomly selected three populations of 20, 50 and 100 sheep for each scenario, with 100 iterations each to estimate the Ne. The Ne was estimated using the same method as for the real population data.

Sargolzaei, M. and F. S. Schenkel. 2009. QMSim: a large-scale genome simulator for livestock. Bioinformatics, 25: 680-681.

**Supplementary Table 2. *Ne* estimates for the simulated populations and a summary of simulation parameters.**

| **QMSim simulation** | | | |
| --- | --- | --- | --- |
| **Populations** | Ne estimate 2400 individuals | Parameters | Demographic effect |
| **POP1** | **184.5** | sd = phen /l | constant |
| **Ne 20** | 199.8 |  |  |
| **Ne 50** | 191.6 |  |  |
| **Ne 100** | 187.9 |  |  |
| **POP2** | **160** | sd = ebv /h | constant |
| **Ne 20** | 169.2 |  |  |
| **Ne 50** | 164.2 |  |  |
| **Ne 100** | 164.9 |  |  |
| **POP2_cd_h** | **163.8** | sd = ebv /h  cd=h | constant |
| **Ne 20** | 177.5 |  |  |
| **Ne 50** | 168.5 |  |  |
| **Ne 100** | 166.8 |  |  |
| **POP3** | **174.3** | sd = phen /l | bottleneck |
| **Ne 20** | 188.9 |  |  |
| **Ne 50** | 186.5 |  |  |
| **Ne 100** | 179.5 |  |  |
| **POP4** | **178.4** | sd = phen /l | expansion |
| **Ne 20** | 186.9 |  |  |
| **Ne 50** | 192.3 |  |  |
| **Ne 100** | 183.4 |  |  |
| **POP5** | **183.8** | sd = rnd | constant |
| **Ne 20** | 194.7 |  |  |
| **Ne 50** | 195.7 |  |  |
| **Ne 100** | 189.5 |  |  |

**Full parameter setting applied in the simulation analyses:**

POP1

/*******************************

** Global parameters **

*******************************/

title = "File 1 - 54k SNP panel";

nrep = 100; //Number of replicates

h2 = 0.15; //Heritability

qtlh2 = 0.005; //QTL heritability

phvar = 1.0; //Phenotypic variance

/*******************************

** Historical population **

*******************************/

begin_hp;

hg_size = 420 [0]; //Size of the historical generations

nmlhg = 20; //Number of males in the last generation

end_hp;

/*******************************

** Populations **

*******************************/

begin_pop = "p1";

begin_founder;

male [n = 20, pop = "hp"];

female [n = 400, pop = "hp"];

end_founder;

ls = 2; //Litter size

md = p_assort /phen;

cd = phen /l;

sd = phen /l;

pmp = 0.5 /fix; //Proportion of male progeny

ng = 10; //Number of generations

begin_popoutput;

data;

stat;

genotype /gen 8 9 10;

ld /gen 8 9 10;

end_popoutput;

end_pop;

/*******************************

** Genome **

*******************************/

begin_genome;

begin_chr = 26;

chrlen = 100; //Chromosome length

nmloci = 2000; //Number of markers

mpos = rnd; //Marker positions

nma = all 2; //Number of marker alleles

maf = rnd; //Marker allele frequencies

nqloci = 3; //Number of QTL

qpos = rnd; //QTL positions

nqa = rnd 2 3 4; //Number of QTL alleles

qaf = rnd; //QTL allele frequencies

qae = rndg 0.4; //QTL allele effects

end_chr;

end_genome;

/*******************************

** Output options **

*******************************/

begin_output;

linkage_map;

end_output;

____________________________________________________

POP2

/*******************************

** Global parameters **

*******************************/

title = "File 1 - 54k SNP panel";

nrep = 100 //Number of replicates

h2 = 0.15; //Heritability

qtlh2 = 0.005; //QTL heritability

phvar = 1.0; //Phenotypic variance

/*******************************

** Historical population **

*******************************/

begin_hp;

hg_size = 420 [0]; //Size of the historical generations

nmlhg = 20; //Number of males in the last generation

end_hp;

/*******************************

** Populations **

*******************************/

begin_pop = "p1";

begin_founder;

male [n = 20, pop = "hp"];

female [n = 400, pop = "hp"];

end_founder;

ls = 2; //Litter size

md = p_assort /phen;

cd = phen /l;

sd = ebv /h;

ebv_est = blup;

pmp = 0.5 /fix; //Proportion of male progeny

ng = 10; //Number of generations

begin_popoutput;

data;

stat;

genotype /gen 8 9 10;

ld /gen 8 9 10;

end_popoutput;

end_pop;

/*******************************

** Genome **

*******************************/

begin_genome;

begin_chr = 26;

chrlen = 100; //Chromosome length

nmloci = 2000; //Number of markers

mpos = rnd; //Marker positions

nma = all 2; //Number of marker alleles

maf = rnd; //Marker allele frequencies

nqloci = 3; //Number of QTL

qpos = rnd; //QTL positions

nqa = rnd 2 3 4; //Number of QTL alleles

qaf = rnd; //QTL allele frequencies

qae = rndg 0.4; //QTL allele effects

end_chr;

end_genome;

/*******************************

** Output options **

*******************************/

begin_output;

linkage_map;

end_output;

_____________________________________________________________

POP2_cd_h

/*******************************

** Global parameters **

*******************************/

title = "File 1 - 54k SNP panel";

nrep = 100; //Number of replicates

h2 = 0.15; //Heritability

qtlh2 = 0.005; //QTL heritability

phvar = 1.0; //Phenotypic variance

/*******************************

** Historical population **

*******************************/

begin_hp;

hg_size = 420 [0]; //Size of the historical generations

nmlhg = 20; //Number of males in the last generation

end_hp;

/*******************************

** Populations **

*******************************/

begin_pop = "p1";

begin_founder;

male [n = 20, pop = "hp"];

female [n = 400, pop = "hp"];

end_founder;

ls = 2; //Litter size

md = p_assort /phen;

cd = phen /h;

sd = ebv /h;

ebv_est = blup;

pmp = 0.5 /fix; //Proportion of male progeny

ng = 10; //Number of generations

begin_popoutput;

data;

stat;

genotype /gen 8 9 10;

ld /gen 8 9 10;

end_popoutput;

end_pop;

/*******************************

** Genome **

*******************************/

begin_genome;

begin_chr = 26;

chrlen = 100; //Chromosome length

nmloci = 2000; //Number of markers

mpos = rnd; //Marker positions

nma = all 2; //Number of marker alleles

maf = rnd; //Marker allele frequencies

nqloci = 3; //Number of QTL

qpos = rnd; //QTL positions

nqa = rnd 2 3 4; //Number of QTL alleles

qaf = rnd; //QTL allele frequencies

qae = rndg 0.4; //QTL allele effects

end_chr;

end_genome;

/*******************************

** Output options **

*******************************/

begin_output;

linkage_map;

end_output;

_____________________________________________________________

POP3

/*******************************

** Global parameters **

*******************************/

title = "File 1 - 54k SNP panel";

nrep = 100; //Number of replicates

h2 = 0.15; //Heritability

qtlh2 = 0.005; //QTL heritability

phvar = 1.0; //Phenotypic variance

/*******************************

** Historical population **

*******************************/

begin_hp;

hg_size = 1000 [0] //Size of the historical generations

200 [70]

200 [80]

420 [100];

nmlhg = 20; //Number of males in the last generation

end_hp;

/*******************************

** Populations **

*******************************/

begin_pop = "p1";

begin_founder;

male [n = 20, pop = "hp"];

female [n = 400, pop = "hp"];

end_founder;

ls = 2; //Litter size

md = p_assort /phen;

cd = phen /l;

sd = phen /l;

pmp = 0.5 /fix; //Proportion of male progeny

ng = 10; //Number of generations

begin_popoutput;

data;

stat;

genotype /gen 8 9 10;

ld /gen 8 9 10;

end_popoutput;

end_pop;

/*******************************

** Genome **

*******************************/

begin_genome;

begin_chr = 26;

chrlen = 100; //Chromosome length

nmloci = 2000; //Number of markers

mpos = rnd; //Marker positions

nma = all 2; //Number of marker alleles

maf = rnd; //Marker allele frequencies

nqloci = 3; //Number of QTL

qpos = rnd; //QTL positions

nqa = rnd 2 3 4; //Number of QTL alleles

qaf = rnd; //QTL allele frequencies

qae = rndg 0.4; //QTL allele effects

end_chr;

end_genome;

/*******************************

** Output options **

*******************************/

begin_output;

linkage_map;

end_output;

____________________________________________________________

POP4

/*******************************

** Global parameters **

*******************************/

title = "File 1 - 54k SNP panel";

nrep = 100; //Number of replicates

h2 = 0.15; //Heritability

qtlh2 = 0.005; //QTL heritability

phvar = 1.0; //Phenotypic variance

/*******************************

** Historical population **

*******************************/

begin_hp;

hg_size = 420 [0] //Size of the historical generations

420 [200];

nmlhg = 20; //Number of males in the last generation

end_hp;

/*******************************

** Populations **

*******************************/

begin_pop = "p1";

begin_founder;

male [n = 20, pop = "hp"];

female [n = 400, pop = "hp"];

end_founder;

ls = 2; //Litter size

md = p_assort /phen;

cd = phen /l;

sd = phen /l;

pmp = 0.5 /fix; //Proportion of male progeny

ng = 10; //Number of generations

begin_popoutput;

data;

stat;

genotype /gen 8 9 10;

ld /gen 8 9 10;

end_popoutput;

end_pop;

/*******************************

** Genome **

*******************************/

begin_genome;

begin_chr = 26;

chrlen = 100; //Chromosome length

nmloci = 2000; //Number of markers

mpos = rnd; //Marker positions

nma = all 2; //Number of marker alleles

maf = rnd; //Marker allele frequencies

nqloci = 3; //Number of QTL

qpos = rnd; //QTL positions

nqa = rnd 2 3 4; //Number of QTL alleles

qaf = rnd; //QTL allele frequencies

qae = rndg 0.4; //QTL allele effects

end_chr;

end_genome;

/*******************************

** Output options **

*******************************/

begin_output;

linkage_map;

end_output;

_____________________________________________________________

POP5

/*******************************

** Global parameters **

*******************************/

title = "File 1 - 54k SNP panel";

nrep = 100; //Number of replicates

h2 = 0.15; //Heritability

qtlh2 = 0.005; //QTL heritability

phvar = 1.0; //Phenotypic variance

/*******************************

** Historical population **

*******************************/

begin_hp;

hg_size = 420 [0]; //Size of the historical generations

nmlhg = 20; //Number of males in the last generation

end_hp;

/*******************************

** Populations **

*******************************/

begin_pop = "p1";

begin_founder;

male [n = 20, pop = "hp"];

female [n = 400, pop = "hp"];

end_founder;

ls = 2; //Litter size

md = p_assort /phen;

cd = phen /l;

sd = rnd;

pmp = 0.5 /fix; //Proportion of male progeny

ng = 10; //Number of generations

begin_popoutput;

data;

stat;

genotype /gen 8 9 10;

ld /gen 8 9 10;

end_popoutput;

end_pop;

/*******************************

** Genome **

*******************************/

begin_genome;

begin_chr = 26;

chrlen = 100; //Chromosome length

nmloci = 2000; //Number of markers

mpos = rnd; //Marker positions

nma = all 2; //Number of marker alleles

maf = rnd; //Marker allele frequencies

nqloci = 3; //Number of QTL

qpos = rnd; //QTL positions

nqa = rnd 2 3 4; //Number of QTL alleles

qaf = rnd; //QTL allele frequencies

qae = rndg 0.4; //QTL allele effects

end_chr;

end_genome;

/*******************************

** Output options **

*******************************/

begin_output;

linkage_map;

end_output;

**(See below) for Supplementary Figures.**

**Workflows**

**
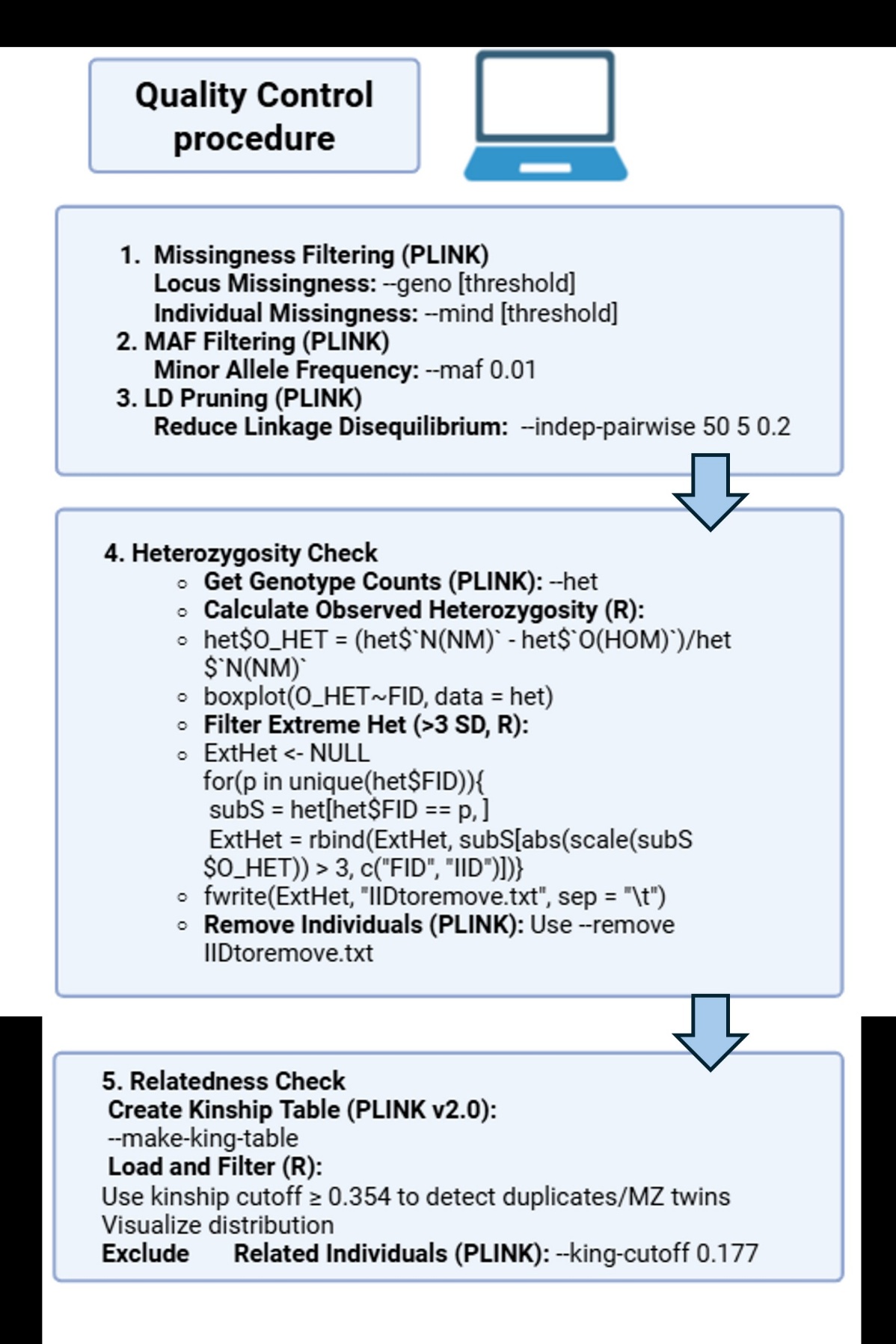
**

Supplementary Figure 1. Quality Control (QC) procedure (adaptation from Ajmone-Marsan et al., 2023).

**
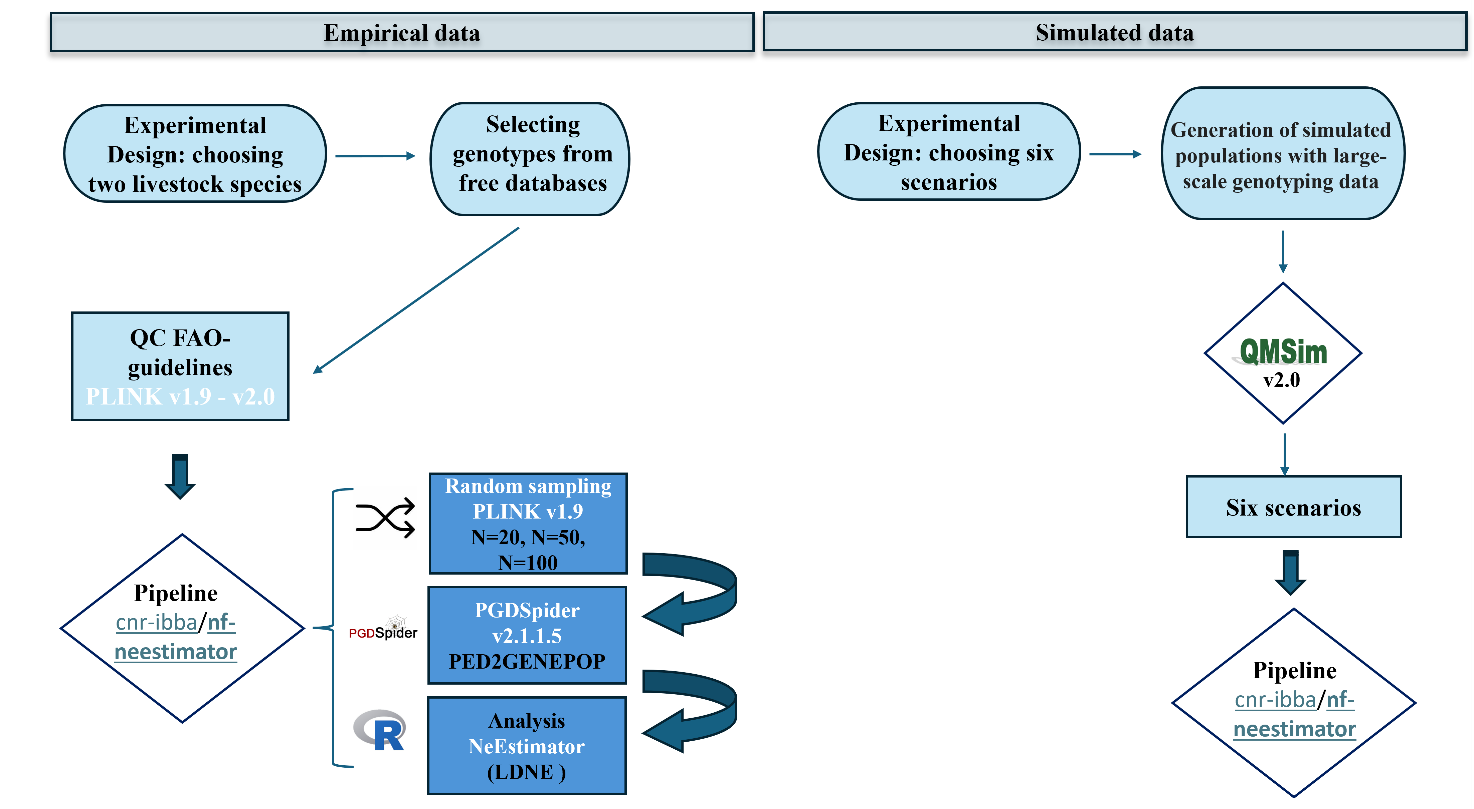
**

Supplementary Figure 2. Workflow for the whole analysis.

**Relationship analysis** (see below).

**
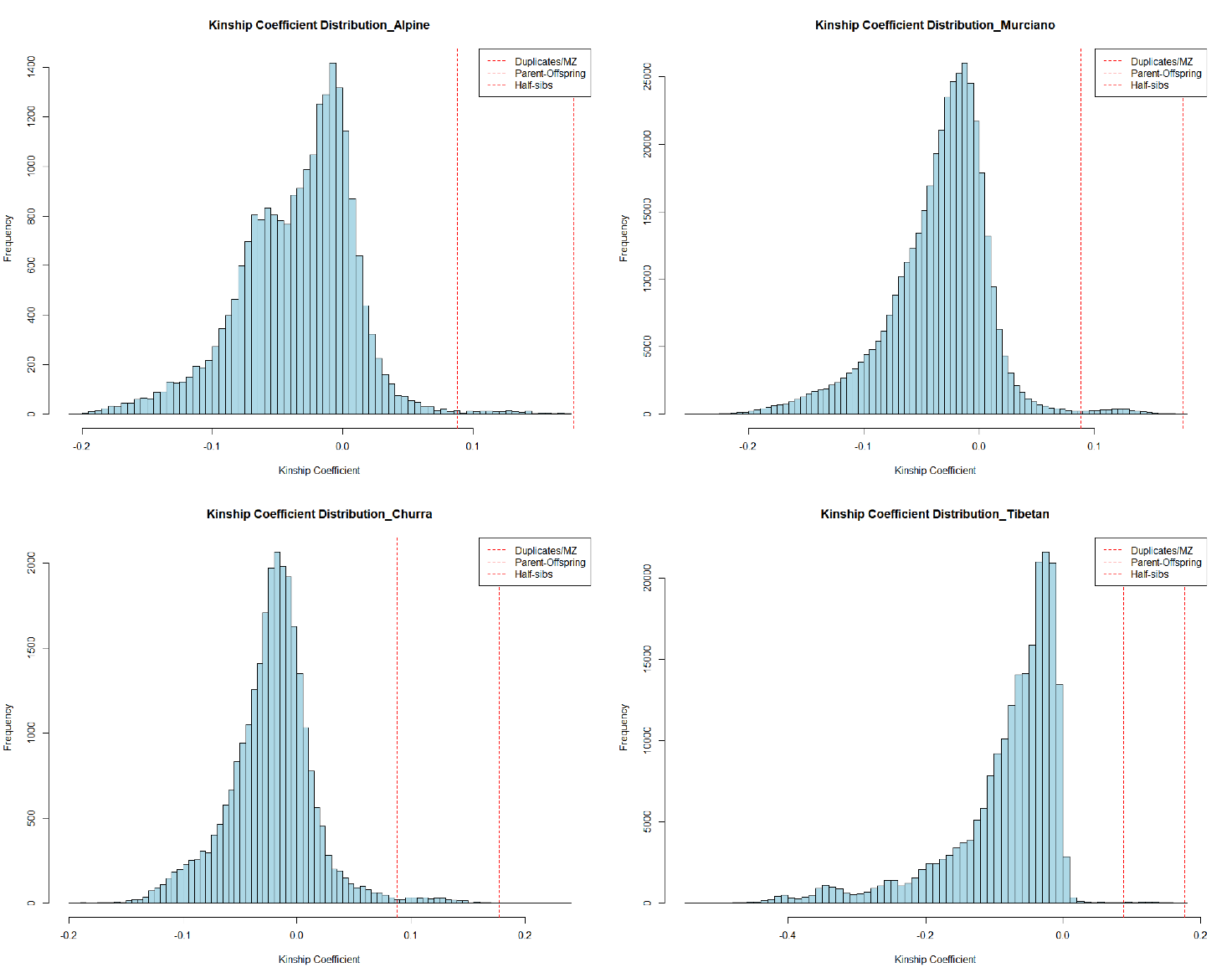
**

Supplementary figure 3. Kinship coefficient distributions across the four breeds. Alpine: moderate skew suggests mild relatedness structure; Murciano-Granadina: the distribution suggests large, well-mixed population (most individuals are unrelated); Churra: this distribution is very similar to that of Murciano-Granadina with not many close relatives. Tibetan: the peak suggests a structured population, with more negative relations in comparison to the other breeds. This is a property of the KING algorithm we applied in the QC when there is population structure (PLINK 2 uses the [KING algorithm](https://www.cog-genomics.org/static/pdf/Manichaikuletal2010.pdf) from Manichaikul et al., 2010).

**
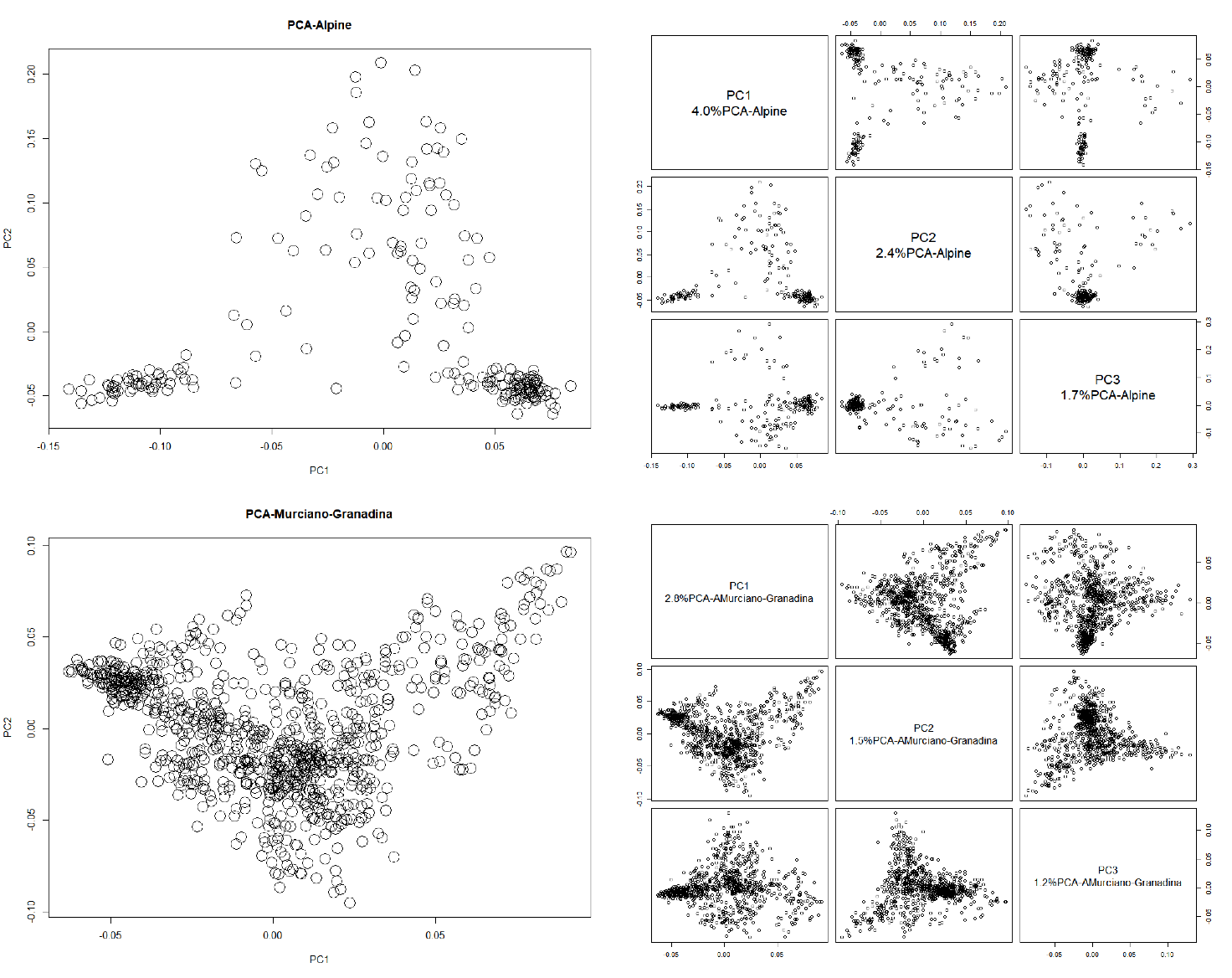
A**

**
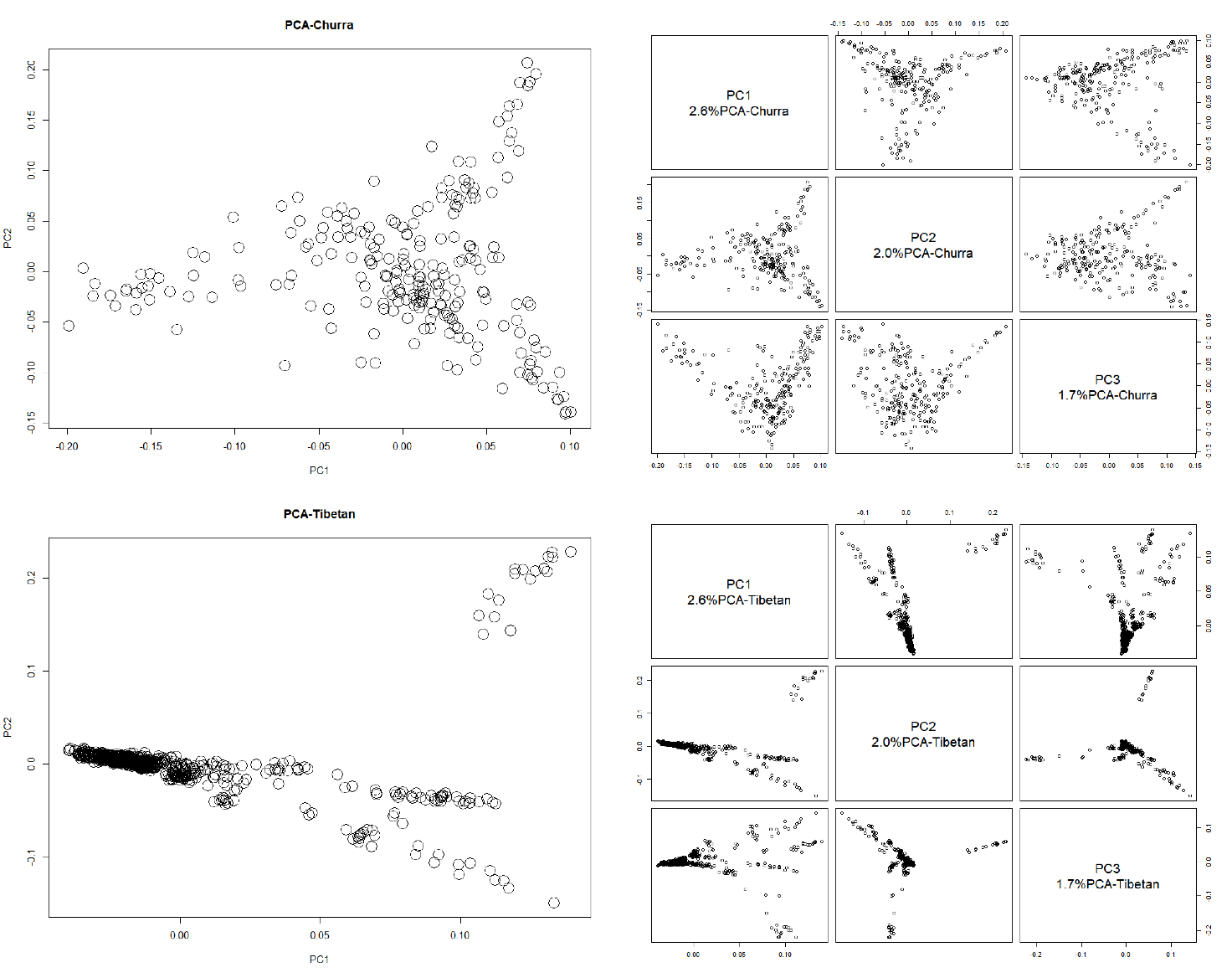
**

**B**

Supplementary Figure 4. PCA plots of the four breeds. A) Alpine and Murciano-Granadina; B) Churra and Tibetan.
